# Chumbos Torácicos e Intramiocárdicos, Achado Incidental em Paciente com Infarto Agudo do Miocárdio

**DOI:** 10.36660/abc.20210854

**Published:** 2022-07-07

**Authors:** Valente Fernandez-Badillo, Mauricio Garcia-Cardenas, Diego Oliva-Cavero, Jose Carlos Armendariz-Ferrari, Erick Alexanderson-Rosas, Nilda Espinola-Zavaleta

**Affiliations:** 1 Instituto Nacional de Cardiologia Ignacio Chávez Departamento de Cardiologia Nuclear Cidade do México México Departamento de Cardiologia Nuclear, Instituto Nacional de Cardiologia Ignacio Chávez, Cidade do México – México; 2 Hospital Nacional Hipólito Unanue Departamento de Cardiologia Lima Peru Departamento de Cardiologia, Hospital Nacional Hipólito Unanue, Lima – Peru

**Keywords:** Chumbo de Caça, Lesão Cardíaca, Arma de Fogo, Infarto Agudo do Miocárdio, Espingarda de Chumbo

## Introdução

O trauma cardíaco penetrante é fatal; aproximadamente mais da metade das pessoas afetadas morrem no local. As feridas penetrantes do miocárdio são raras e a retenção de chumbos cardíacos é pouco documentada na literatura.^1^ Não existem protocolos padronizados para sua abordagem diagnóstica e terapêutica até o momento. A apresentação clínica de uma lesão por espingarda depende do tamanho da ferida, do local de entrada e da lesão dos grandes vasos.^2^ No trauma torácico penetrante, ambos os ventrículos são lesados com frequência semelhante, mas o ventrículo direito é o local de maior entrada porque forma a maior parte da face anterior do coração.^3^

### Apresentação do caso

Apresentamos o caso de um homem de 59 anos com história familiar de hiperlipidemia e infarto agudo do miocárdio (IAM) e história pessoal de trauma torácico secundário a ferimento por espingarda em 2006, que não mereceu tratamento cirúrgico, e diabetes mellitus tipo 2 diagnosticado em 2016 em tratamento médico com sitagliptina. O paciente chegou ao pronto-socorro em janeiro de 2018 com dor torácica opressiva de 6 horas de evolução, intensidade 8/10, irradiação para o braço esquerdo e sudorese. À admissão, os sinais vitais estavam dentro dos parâmetros normais, com pressão arterial -120/70 mmHg, frequência cardíaca -75 bpm, saturação de oxigênio -92% e índice de massa corporal -26 kg/m^2^.

**Figura 1:**
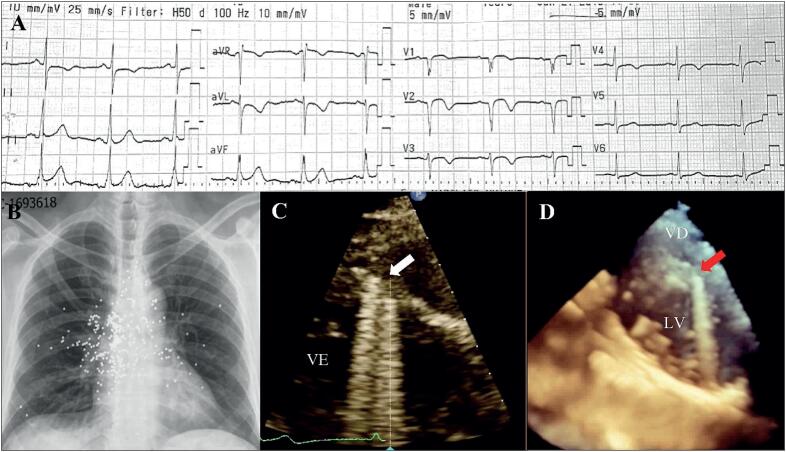
Diagnóstico por imagem multimodal. (A) Eletrocardiograma de 12 derivações com ritmo sinusal, 73 bpm, onda Q nas derivações V1-V4 com supradesnivelamento do segmento ST e inversão da onda T, sugerindo isquemia da parede ântero-septal. (B) Radiografia de tórax em anteroposterior com inúmeros objetos circulares radiopacos, densidade metálica. (C) 2D-TTE com imagem hipoecoica no segmento médio do septo interventricular (seta) com realce posterior. (D) 3D-TTE, semelhante aos achados da figura 1C. VE: ventrículo esquerdo; VD: ventrículo direito.

**Figura 2:**
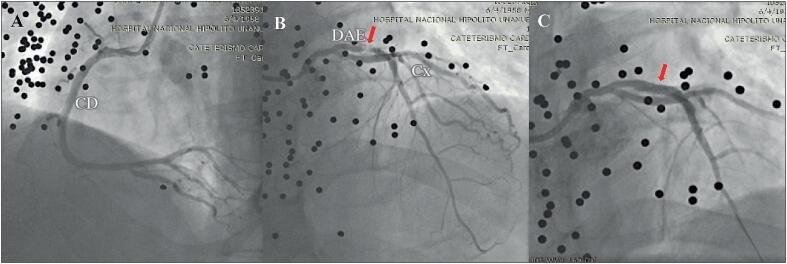
Cateterismo cardíaco. Presença de inúmeros objetos circulares compatíveis com chumbos. (A) Artéria coronária direita normal. (B) Artéria descendente anterior esquerda com obstrução de 95% no segmento médio (seta). (C) Implante de stent da artéria coronária descendente anterior esquerda (seta), fluxo TIMI III com sucesso. Cx: circunflexo; DAE: descendente anterior esquerda; CD: coronária direita.

**Figura 3:**
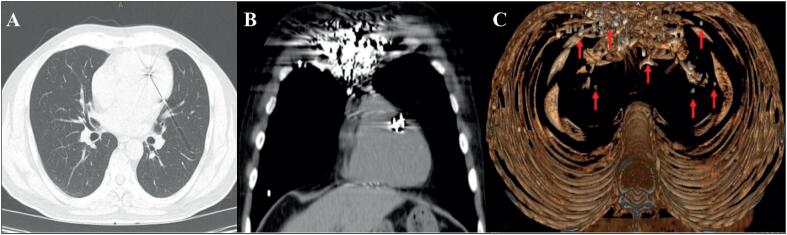
Tomografia computadorizada de tórax. (A,B). 2D-TC com objetos esféricos hiperintensos no mediastino, parede torácica anterior e coração, aparentemente no átrio esquerdo. (C) reconstrução 3D, chumbos no mediastino e intramiocárdico (setas).

Ao exame físico, apresentava cicatriz queloide antiga na região torácica anterior, batimento apexiano hiperdinâmico no quinto espaço intercostal esquerdo, não sendo detectados sopros cardíacos ou ruídos pulmonares abdominais. O eletrocardiograma mostrava ritmo sinusal, frequência cardíaca -73 bpm, onda Q nas derivações V1 a V4 com supradesnivelamento do segmento ST e inversão da onda T nas mesmas derivações (Figura 1A). Os exames laboratoriais mostraram leucocitose (13,06 x109/L), fibrinogênio elevado (638 g/L), hipocalemia (3,3 mEq/L), hiperglicemia (250 mg/dL), HbAC1-8,7%, marcadores positivos de dano miocárdico (CPK-411 UI/L, CPK-MB-53 ng/mL e troponina de alta sensibilidade-6,1 ng/dL), hipercolesterolemia (colesterol total-256 mg/dL, c-HDL-35 mg/dL e c-LDL-186 mg /dL) e hipertrigliceridemia (278 mg/dL). O ecocardiograma transtorácico (ETT) bidimensional mostrou volume ventricular e fração de ejeção do ventrículo esquerdo (FEVE) normais de 67%, disfunção diastólica tipo II e imagem hipoecoica no segmento médio do septo interventricular com realce posterior (Figura 1C-D). O cateterismo cardíaco mostrou obstrução de 95% do segmento médio da artéria descendente anterior, sendo necessária angioplastia com balão para obtenção de fluxo TIMI III; surpreendentemente, foram observados inúmeros objetos esféricos compatíveis com chumbos em todas as regiões cardíacas (Figura 2). A radiografia de tórax póstero-anterior revelou múltiplos objetos circulares radiopacos com predomínio na região anterior do tórax (Figura 1B). A tomografia computadorizada (TC) de tórax 2D mostrou múltiplos objetos esféricos hiperintensos no mediastino, parede torácica anterior, (Figura 3).

O paciente recebeu alta 3 dias depois, hemodinamicamente estável, sem necessidade de intervenção cirúrgica e optou-se por tratamento conservador devido à ausência de sintomas ou complicações cardiovasculares após 12 anos de trauma cardíaco. Os acompanhamentos foram agendados a cada 3 meses no ambulatório de cardiologia, sendo indicadas mudanças no estilo de vida e tratamento medicamentoso com antiplaquetários, estatinas e hipoglicemiantes orais. Atualmente, 42 meses após o seguimento, o paciente encontra-se em classe funcional I da NYHA.

## Discussão

A violência armada é um grave problema de saúde pública, que causa a morte de mais de 250.000 pessoas por ano em todo o mundo. Guenther et al.,^4^ identificaram até 2020, 40 casos notificados de lesões cardíacas causadas por espingarda de chumbo. Destes, 90% eram homens, com idade média de 14 anos; 48% dos pacientes foram relatados hemodinamicamente instáveis. Uma esternotomia foi realizada em 58% dos casos, circulação extracorpórea em 18% e janela pericárdica em 15%. Os principais locais acometidos foram ventrículo direito em 43%, ventrículo esquerdo em 33%, átrio direito em 15% e átrio esquerdo e grandes vasos foram acometidos em 6%, respectivamente.^5^ As complicações incluem embolização pela injeção (25%), morte (13%), hemorragia maciça, tamponamento cardíaco, dano direto à parede livre dos ventrículos ou septo interventricular, dissecção de artérias coronárias e lesão do sistema de condução.^4–6^ O trauma cardíaco é um dos fatores de risco associados ao aparecimento do infarto agudo do miocárdio; entretanto, os casos relatados são isolados.^2–5^

A TC e a ecocardiografia são comumente os exames de imagem mais utilizados para diagnosticar lesões cardíacas traumáticas. O ETT bidimensional é o método mais preciso para identificar lesões cardíacas, enquanto a TC é o melhor para localizar corpos estranhos. A detecção de corpos estranhos intracavitários é uma indicação de sua remoção cirúrgica devido ao alto risco de desenvolver eventos trombóticos, enquanto a presença de corpos estranhos completamente intramiocárdicos é mais indicativa de conduta conservadora.^1,2,4^

## Conclusão

A retenção de chumbos intramiocárdicos sem sintomas é uma condição rara no trauma torácico, sendo isolados os casos associados ao infarto agudo do miocárdio. Não existem diretrizes padronizadas para a abordagem diagnóstica e de manejo desse tipo de lesão, provavelmente devido ao baixo número de casos notificados. Além disso, destacamos o uso da imagem multimodal como ferramenta inestimável para o diagnóstico preciso desse tipo de lesão.
