# Supplementary material for: Chumbos Torácicos e Intramiocárdicos, Achado Incidental em Paciente com Infarto Agudo do Miocárdio
Source: Arq Bras Cardiol. 2022 Jul 7;119(1):133–5. [Article in Portuguese] doi: 10.36660/abc.20210854 (PMC9352119; doi:10.36660/abc.20210854)
Supplement: Translation

# Translation: Thoracic and Intramyocardial Pellets, an Incidental Finding in a Patient with Acute Myocardial Infarction

**Authors:** Valente Fernandez-Badillo, Mauricio Garcia-Cardenas, Diego Oliva-Cavero, Jose Carlos Armendariz-Ferrari, Erick Alexanderson-Rosas, Nilda Espinola-Zavaleta

**Affiliations:** 1 Ciudad de México Mexico Departamento de Cardiologia Nuclear, Instituto Nacional de Cardiologia Ignacio Chávez, Ciudad de México – México; 2 Lima Peru Departamento de Cardiologia, Hospital Nacional Hipólito Unanue, Lima – Peru

**Keywords:** Myocardial Infarction/diagnostic, imaging, Wounds, Gunshot, Myocardial Contusion, Incidental Findings, Firearms, Lead

## Introduction

Penetrating cardiac trauma is fatal; approximately more than half of affected people die at the scene. Penetrating myocardial wounds are rare, and the retention of cardiac pellets is poorly documented in literature.^1^ There are no standardized protocols for their diagnostic and therapeutic approach until now. Clinical presentation of a shotgun injury depends on the wound size, entry site, and the injury to the great vessels.^2^ In penetrating chest trauma, both ventricles are injured with similar frequency, but the right ventricle is the most entry site because it forms most of the anterior surface of the heart.^3^

### Case presentation

We present the case of a 59-years-old man with a family history of hyperlipidemia and acute myocardial infarction (AMI) and a personal history of chest trauma secondary to a shotgun injury in 2006, which did not deserve surgical treatment, no more event data, and type 2 diabetes mellitus diagnosed in 2016 under medical treatment with sitagliptin. The patient arrived at the emergency room in January 2018 with oppressive chest pain of 6 hours of evolution, intensity 8/10, radiated to the left arm, and diaphoresis. At admission, vital signs were within normal parameters, with blood pressure-120/70 mmHg, heart rate-75 bpm, oxygen saturation-92% and body mass index-26 kg/m^2^.

Physical examination revealed an old keloid scar in the anterior thoracic region, a hyperdynamic apexian beat in the fifth left intercostal space, and no heart murmurs or abdominal lung sounds were detected. The electrocardiogram showed sinus rhythm, heart rate-73 bpm, Q wave in V1 to V4 leads with ST-segment elevation and inversion of the T wave in the same leads (Figure 1A). Laboratory tests showed leukocytosis (13.06 x10^9^/L), elevated fibrinogen (638 g/L), hypokalemia (3.3 mEq/L), hyperglycemia (250 mg/dL), HbAC1-8.7%, positive markers of myocardial damage (CPK-411 IU/L, CPK-MB-53 ng/mL, and high-sensitive troponin-6.1 ng/dL), hypercholesterolemia (total cholesterol-256 mg/dL, c-HDL-35 mg/dL and c-LDL-186 mg/dL) and hypertriglyceridemia (278 mg/dL). A two-dimensional transthoracic echocardiogram (TTE) showed normal ventricular volume and left ventricular ejection fraction (LVEF) of 67%, type II diastolic dysfunction, and a hypoechoic image in the middle segment of the interventricular septum with posterior enhancement (Figure 1C-D). Cardiac catheterization showed a 95% obstruction in the middle segment of the left anterior descending artery, which required balloon angioplasty to obtain TIMI III flow; surprisingly, were observed countless spherical objects compatible with pellets in all cardiac regions (Figure 2). The posteroanterior chest X-ray revealed multiple radiopaque circular objects with a predominance in the anterior region of the thorax (Figure 1B). The 2D chest computed tomography (CT) showed multiple hyperintense spherical objects in the mediastinum, anterior thoracic wall, and the heart, apparently in the left atrium (LA) and the 3D-reconstruction CT confirmed the presence of intramyocardial pellets (Figure 3).

**Figure 1:** Multimodal imaging diagnosis. (A) 12-leads electrocardiogram with sinus rhythm, 73 bpm, Q wave in V1-V4 leads with ST-segment elevation and T wave inversion, suggesting anteroseptal wall ischemia. (B) Anteroposterior chest x-ray with uncountable radiopaque circular objects, metal density. (C) 2D-TTE with a hypoechoic image in the middle segment of the interventricular septum (arrow) with posterior enhancement. (D) 3D-TTE, similar to findings of figure 1C. LV: left ventricle; RV: right ventricle.

**Figure 2:** Cardiac catheterization. Presence of uncountable circular objects compatible with pellets. (A) Normal right coronary artery. (B) Left anterior descending artery with obstruction of 95% in the middle segment (arrow). (C) Successful left anterior descending coronary artery stenting (arrow), TIMI III flow. Cx: circumflex; LAD: Left anterior descending; RC: right coronary.

**Figure 3:** Chest computed tomography. (A, B). 2D-CT with hyperintense spherical objects in the mediastinum, anterior thoracic wall, and the heart, apparently in the left atrium. (C) 3D-reconstruction, pellets in mediastinum and intramyocardial (arrows).

The patient was discharged 3 days later, hemodynamically stable, no surgical intervention was required, and conservative treatment was chosen due to the absence of cardiovascular symptoms or complications after 12 years of cardiac trauma. Follow-ups were scheduled every 3 months in the cardiology outpatient clinic, and changes in lifestyle and drug treatment with antiplatelet agents, statins and oral hypoglycemic agents were indicated. Currently, 42 months after follow-up, the patient is in NYHA functional class I.

## Discussion

Gun violence is a serious public health problem, which causes the death of more than 250,000 people by year worldwide. Guenther and collaborators^4^ identified up to 2020, 40 reported cases of cardiac injuries caused by a pellet gun. Of these, 90% were men, with an average age of 14 years old; 48% of the patients were reported hemodynamically unstable. Sternotomy was performed in 58% of the cases, a cardiopulmonary bypass in 18% and a pericardial window in 15%. The main affected sites were the right ventricle in 43%, the left ventricle in 33%, the right atrium in 15%, and the left atrium and great vessels were affected in 6%, respectively.^5^ Complications include embolization caused by the shot (25%), death (13%), massive hemorrhage, cardiac tamponade, direct damage to the free wall of ventricles or interventricular septum, dissection of coronary arteries and damage to the conduction system.^4–6^ Cardiac trauma is one of the risk factors associated with the appearance of acute myocardial infarction; however, reported cases are isolated.^2–5^

CT and echocardiography are commonly the most used imaging studies to diagnose traumatic cardiac injuries. Two-dimensional TTE is the most accurate method for identifying cardiac lesions, whereas CT is the best for locating foreign bodies. The detection of intracavitary foreign bodies is an indication of their surgical removal due to the high risk of developing thrombotic events, while the presence of completely intramyocardial foreign bodies is more indicative of conservative management.^1,2,4^

## Conclusion

Retention of intramyocardial pellets without symptoms is a rare condition in thoracic trauma, and cases associated with acute myocardial infarction are isolated. There are no standardized guidelines for this type of injury’s diagnostic and management approach, probably due to the low number of reported cases. Also, we emphasize the use of multimodal imaging as an invaluable tool for the accurate diagnosis of this type of injury.
